# Stability and uptake of methylphenidate and ritalinic acid in nine-spine stickleback (*Pungitius pungitius*) and water louse (*Asellus aquaticus*)

**DOI:** 10.1007/s11356-019-04557-9

**Published:** 2019-02-25

**Authors:** Erin S. McCallum, Richard H. Lindberg, Patrik L. Andersson, Tomas Brodin

**Affiliations:** 1 Department of Ecology and Environmental Science, Umeå University, 901 87 Umeå, Sweden; 2 Present Address: Department of Wildlife, Fish, and Environmental Studies, Swedish University of Agricultural Sciences (SLU), 901 83 Umeå, Sweden; 3 Department of Chemistry, Umeå University, 901 87 Umeå, Sweden

**Keywords:** Pharmaceuticals, Ecotoxicology, Stimulant, Behaviour, Methylphenidate, Aquatic organisms

## Abstract

The presence of human pharmaceuticals in the environment has garnered significant research attention because these compounds may exert therapeutic effects on exposed wildlife. Yet, for many compounds, there is still little research documenting their stability in the water column and uptake in organism tissues. Here, we measured the uptake and stability of methylphenidate (Ritalin®, a frequently prescribed central nervous system stimulant) and its primary metabolite, ritalinic acid, in (1) water only or (2) with nine-spine stickleback and water louse. Methylphenidate degraded to ritalinic acid in both studies faster at a higher temperature (20 °C versus 10 °C), with concentrations of ritalinic acid surpassing methylphenidate after 48–100 h, depending on temperature. The concentration of methylphenidate in stickleback was highest at the first sampling point (60 min), while the concentration in water louse tissues reached comparatively higher levels and peaked after ~ 6 days. Neither stickleback nor water louse took up ritalinic acid in tissues despite being present in the water column. Our findings provide valuable data for use in future risk assessment of methylphenidate and will aid in the design of studies aimed at measuring any ecotoxicological effects on, for example, the behaviour or physiology of aquatic organisms.

**Electronic supplementary material:**

The online version of this article (10.1007/s11356-019-04557-9) contains supplementary material, which is available to authorized users.

## Introduction

The presence of pharmaceuticals in the environment has drawn widespread concern from researchers and environmental managers over the impacts that exposure to pharmaceuticals might have on, for example, antibiotic resistance and aquatic wildlife (Boxall et al. 2012; Brodin et al. 2014). Many pharmaceutical compounds are present in the environment, but attention on compounds that modulate human behaviour—such as antidepressants, anxiolytics, or stimulants—has recently grown (Calisto and Esteves 2009; Brodin et al. 2014; Saaristo et al. 2018). The biological targets of many pharmaceuticals are shared between humans and aquatic vertebrates (Gunnarsson et al. 2008). Therefore, unintended environmental exposure to pharmaceuticals in the wild may cause subtle changes to animal behaviour (e.g., predator avoidance, reproduction) that could have important and potentially detrimental effects on fitness in the wild.

One such compound with the potential to modulate behaviour is methylphenidate (e.g., Ritalin®, Concerta®): a central nervous system stimulant prescribed to promote executive functions such as attention and focus in humans. Methylphenidate is a commonly prescribed treatment for attention deficit hyperactivity disorder (ADHD), and the non-medical, recreational use of methylphenidate has been increasing (McCabe et al. 2005; Chai et al. 2012). Methylphenidate increases dopaminergic and norepinephric transmission via inhibiting the reuptake of these catecholamines from the synaptic cleft (Volkow et al. 2002). Following a standard dose (18 mg), up to 80% is excreted as ritalinic acid (α-phenyl-2-piperidine acetic acid) that has little pharmacological activity (Faraj et al. 1974; Concerta Prescribing Information 2013). Recently, Endres et al. (2017) showed that exposure to environmentally relevant concentrations of methylphenidate reduced cortisol (i.e., stress) and inhibited anxiety-like behaviours (e.g., freezing, hiding) in stressed zebrafish (*Danio rerio*). Reductions in anxiety behaviours have been noted in adult guppies (*Poecilia reticulata*) exposed to 250 ng/L (De Serrano et al. 2016) and in zebrafish exposed to a much higher concentration (50 mg/L) as embryos (Levin et al. 2011).

Methylphenidate has been detected in treated wastewater effluents at low concentrations (< 10 ng/L), and this is, in part, because it is readily transformed before excretion (Letzel et al. 2010; van der Aa et al. 2013; Du et al. 2014; Watkins et al. 2014; Bean et al. 2018). However, in untreated wastewater, levels up to 1500 ng/L have been found (Burgard et al. 2013). The primary metabolite, ritalinic acid has been detected at concentrations ranging from 50 to 300 ng/L in treated effluent (Letzel et al. 2010; Burgard et al. 2013; Oliveira et al. 2015). Methylphenidate and ritalinic acid have low octanol-water partitioning coefficients; methylphenidate (0.20) and ritalinic acid (− 1.1), and uptake occurs mainly from the water phase (US EPA EPISuite n.d.). Their predicted bioconcentration factors are, however, very low (3.2), which indicates low expected levels of these compounds in aquatic biota (US EPA EPISuite n.d.).

Given recent and growing research attention on methylphenidate and ritalinic acid as environmental pollutants, more detailed investigations of their stability in the water column and concentrations in the tissues of aquatic organisms are needed. Basic data on the stability of compounds in water and uptake in tissue is needed to effectively design laboratory tests. To support future studies on effects on aquatic organisms, we measured concentrations of methylphenidate and ritalinic acid in the water column under static laboratory conditions, with and without the presence of biota over 2 weeks. In our biota study, we measured the tissue uptake of both compounds in muscle tissue of the nine-spine stickleback (*Pungitius pungitius*) and whole-body homogenates of water louse (*Asellus aquaticus*) to compare uptake in a vertebrate and invertebrate. Both species are widespread in temperate freshwater environments in North America, Europe, and Asia, making these results ecologically relevant and widely applicable. We included a temperature treatment (10 °C and 20 °C) in this study to test how temperature affects the degradation of both compounds in the exposure environment and uptake in tissues.

## Methods

### Preparation of stock solutions

Stock solutions were prepared by dissolving methylphenidate hydrochloride (CAS 298-59-9; Merck Darmstadt, Germany) in crystalline form in Milli-Q ultra-pure water (Millipore advantage A10, Billerica, USA), acidified with 0.1% formic acid (Suprapur, Merck KGaA), and mixed for 5 min using an ultrasonic bath. The pKa of methylphenidate is 9.5 (SciFinder), and the stock solution was acidified to a pH of ~ 2.7 to keep the compound in the neutral form. The experiments were conducted at ~ pH 7 conditions, which means that the level of the ionized form is negligible. The stock solution for study 1 (water only) was prepared on December 15, 2017, and the exposure began the next day. For study 2 (water and biota), the stock solution was prepared on January 29, 2018, and the exposures began on the same day.

### Study 1: static water only

The stability of methylphenidate and breakdown to ritalinic acid was first measured in water without biota. A total of 1870 ng/L (nominal concentration) of methylphenidate (1 mL of the stock solution) was added to a glass aquarium filled with 20 L of ground water. The tank was aerated and kept at 20 °C (ambient room temperature). Water was sampled at 0 min (immediately after dosing), 5 min, 15 min, 45 min, 90 min, 4 h, 10 h, 24 h, 2 days, 3 days, 4 days, 5 days, 7 days, and 12 days. This exposure was conducted in a windowless room with a light-dark cycle of 13L:11D.

### Study 2: static water + biota

Nine-spine stickleback were collected using umbrella traps from ponds in Röbäck, Sweden, in July 2017. Fish were housed in a large, aerated, ground water flow-through tank (1500 L) until the start of the experiment. Fish were fed frozen chironomid larvae until satiation once daily. Water louse were collected using sweep nets from Djupsundsbäcken near Holmsund, Sweden, in January 2018. Water louse were kept in aerated containers with detritus (predominately alder leaf litter) until the start of the experiment.

Stickleback and water louse were exposed to 187 ng/L (nominal concentration) methylphenidate at two temperature treatments: 10 °C (in a climate chamber) and 20 °C (ambient room temperature). Both exposures were maintained at the same light cycle used in study 1. Organisms were exposed under static conditions using three replicate glass aquaria filled with 20 L water. The aquaria were equipped with an airstone and two ceramic clay pots for shelter in each temperature treatment. Fish were exposed in two tanks, while water louse were exposed separately in the remaining tank. Water from each tank was sampled at 0 min (immediately after dosing), 15 min, 30 min, 60 min, 4 h, 8 h, 24 h, 3 days, 5 days, 7 days, 9 days, 10 days, 11 days, 12 days, and 13 days. Three to six water louse and three to six fish were sampled at each of the following time points: 60 min, 4 h, 8 h, 24 h, 3 days, 5 days, 7 days, 9 days, 10 days, 11 days, 12 days, and 13 days. Fish were euthanized via cerebral concussion and spinal severance. Water quality measures of pH (Merck pH Universal Indicator Strips), dissolved oxygen, and temperature (YSI Pro DO Series) were verified in the exposure tanks on the final days of sampling. For the low (10 °C) and high (20 °C) temperature rooms, respectively, water temperature was (mean ± s.d.) 10.03 ± 0.32 °C and 17.07 ± 0.21 °C; dissolved oxygen was 14.40 ± 0.27 mg/L and 12.30 ± 0.30 mg/L; and there were no changes in pH (7 ± 0, for both temperatures).

### Chemical analysis

Immediately after sampling, all water and organisms sampled were frozen at − 20 °C until further processing. Muscle tissue (mean ± s.d. 0.09 ± 0.02 g; range 0.02–0.13 g) was dissected from each stickleback by taking a section of the dorsal axial muscle. Water louse collected from each temperature treatment (10 °C and 20 °C) on each sampling day were pooled due to low sample weight before extracting whole-body tissue (0.05 ± 0.01 g; range 0.03–0.07 g). However, when enough tissue was available, multiple water louse samples were analysed. To each sample, 5 ng (50 μL) of the internal standard methylphenidate-D9 (Cerilliant, USA; in methanol, HPLC-grade, Fisher Chemical, Loughborough, UK) was added. For both stickleback and water louse, tissues were extracted using 1.5 ml acetonitrile (HPLC-grade, Fisher Chemical, Loughborough, UK) repeated twice (based on the method used by Brodin et al. 2013; McCallum et al. 2017, 2018). The samples were homogenized and extracted for 4 min at 42,000 oscillations per minute (Mini Beadbeater, Biospec. Bartlesville, USA) with zirconium beads and then centrifuged the samples at 17,500 g for 10 min (Beckman Coulter Microfuge 22R Centrifuge). This protocol was used for both eluent mixtures, and the supernatants were combined. The combined supernatants were evaporated to 20 μl and the sample was reconstituted in 100 μl methanol (HPLC-grade, Fisher Chemical, Loughborough, UK).

Fifty microlitres of methylphenidate-D9 (500 ng/L) was added to 10-ml aliquots of the aqueous samples, filtered (0.45 μm, Filtropur S, Sarstedt, N~umbrecht, Germany), and acidified with 10 μL formic acid (0.1% *v*/*v*, Merck KGaA, Darmstadt, Germany) to a pH of 2.7.

All of the samples were injected to an EQuan MAX Plus LC-Quantiva triple quadrupole MS/MS (Thermo Scientific, San Jose, USA). A 10-μL injection volume was used for stickleback/louse extracts and the calibration curve. For the aqueous samples, and the corresponding calibration curve, a 1-mL injection volume was used and preconcentrated the analytes on an online-connected Oasis HLB column (2.1 × 20 mm, 15 μm Waters, Milford, USA) using column switching (70 s transfer time and 180 s elution time). Milli-Q ultra-pure water (Millipore) and methanol (Lichrosolv, Hypergrade, Merck) were used as mobile phases (both containing 0.1% formic acid (Suprapur, Merck KGaA), *v*/*v*) together with Hypersil GOLD columns (Thermo Scientific) as stationary phases: guard (20 × 2.1 mm, 3 μm) and analytical (50 × 2.1 mm, 3 μm). The flow rate was kept at 350 μL/min, and we used two linear gradients. The gradient for the stickleback/louse extracts were as follows: 0–2 min, methanol 2%; 2–4 min, methanol 2–100%; 4–6 min, methanol 100%; and 6.01–7.5 min, methanol 2%. For the aqueous samples, the gradient was 0–2 min, methanol 2%; 2–4.75 min, methanol 2–100%; 4.75–7 min, methanol 100%; and 7.01–9 min, methanol 2%.

Positive polarity and a heated electrospray at 338 °C, an ion transfer tube temperature at 350 °C, and a spray voltage at 3.5 kV were used. The following selected reaction-monitoring (SRM) transitions for quantification ions (Q) and qualitative (q) ions were used: methylphenidate 234.14 → 84.11 (Q), 56.18 (q); ritalinic acid 220.14 → 84.11 (Q), 174.18 (q); and methylphenidate-D9 243.2 → 93.17 (Q), 61.17 (q). A 9-point calibration curve was used from 0.05 to 1000 ng/g or ng/L (*r*^2^ > 0.99 for both compounds). The first or the second point within the linear range was used for the limits of quantification (LOQ): methylphenidate 1 ng/L (aqueous) and 0.1 ng/g (biota); and ritalinic acid 1 ng/L (aqueous) and 1 ng/g (biota). See supplementary materials for QA/QC data.

### Statistical analyses

All analyses were conducted in R (version: 3.5.0; R Core Team 2018). The average concentrations of methylphenidate and ritalinic acid in the water column and the uptake of both compounds in tissues were descriptively summarized. Welch’s two-sample *t* tests were used to analyse whether methylphenidate degradation in the water column was impacted by temperature treatment. The half-life of methylphenidate and formation rate of ritalinic acid in water was calculated by plotting the natural log of the concentrations measured versus time of sampling. Degradation/formation rate, *k*_d_, is the slope given by a linear regression analysis. Half-life was determined using the following equation:

1 $$ {t}_{1/2}\  or\ {t}_f=\frac{\ln (2)}{k_d} $$

in which *t*_1/2_ equals the half-life of methylphenidate and *t*_f_ equals the formation of ritalinic acid (d) and *k*_d_ equals the depuration rate. Relative tissue concentration for both compounds in the stickleback and water louse tissues was calculated using the following equation:

2 $$ Relative\ tissue\ concentration=\frac{C_b}{C_w} $$

where *C*_b_ is the methylphenidate/ritalinic acid concentration measured in biota and *C*_w_ is the methyl phenidate/ritalinic acid concentration measured in the exposure water. Relative tissue uptake was calculated at each sampling point.

## Results

### Fate of methylphenidate and ritalinic acid in the water column

**Fig. 1:**
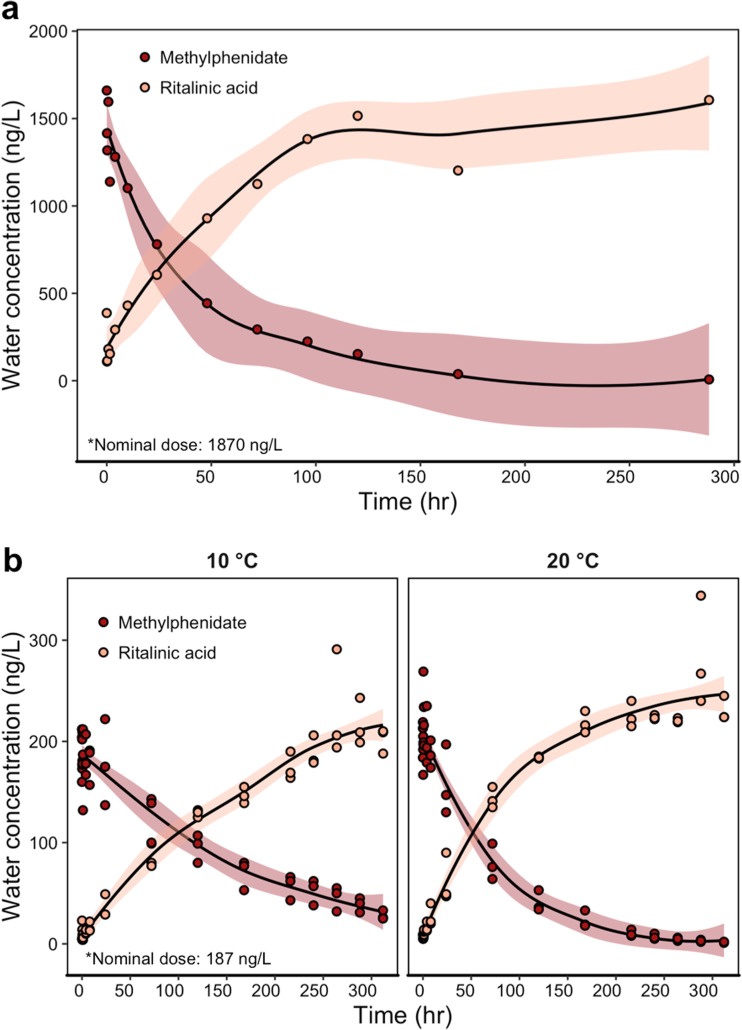
Concentrations of methylphenidate and ritalinic acid in exposure aquaria under (**a**) static conditions with no biota (*N*_tanks_ = 1) and (**b**) static conditions with biota, facetted by temperature treatment (*N*_tanks_ = 3). Each point represents one sample. Solid lines represent the mean fit by a loess curve, with shaded ribbons showing the 95% confidence interval of the mean

Methylphenidate quickly hydrolysed to ritalinic acid (Fig. 1a), with ritalinic acid concentrations surpassing methylphenidate concentrations between the 48 and 100 h in both studies (i.e., water without biota and with biota). Methylphenidate degraded to ritalinic acid and faster at 20 °C than at 10 °C; likewise, ritalinic acid formed faster at 20 °C than at 10 °C (Table 1; Fig. 1b). In both studies, methylphenidate was still detectable in the water column above the LOQ after 2 weeks (Table 1). Temperature did not affect the concentration of methylphenidate immediately after dosing (Fig. 1b, Table 1, Welch two-sample *t* test, *t*_3.50_ = − 0.69, *p* = 0.54), but on the final day of sampling, the concentration of methylphenidate was higher in 10 °C tanks than in 20 °C tanks (Fig. 1b, Table 1: Welch two-sample *t* test, *t*_2.07_ = 10.37, *p* = 0.0081). Concentrations of ritalinic acid did not differ between temperature treatments at the start of the experiment (Welch two-sample *t* test, *t*_2.20_ = 0.54, *p* = 0.64) but were higher in 20 °C tanks than in 10 °C tanks at the end of the experiment (Welch two-sample *t* test, *t*_4.00_ = − 2.86, *p* = 0.046). See Supplementary Materials Fig. S1ab for increased resolution of sampling across the first 24 h.

**Table 1:** Water concentrations (ng/L) of methylphenidate and ritalinic acid from study 1 and study 2, with the half-life or formation rate for methylphenidate or ritalinic acid, respectively. When appropriate, data are shown as mean ± s.d. Start sampling occurred immediately after dosing. End sampling occurred on the final sampling day. – indicates no data, and tanks were only dosed with methylphenidate

	Nominal	Measured start	Measured end	Half-life or formation time (h)
Study 1: static water only, *N*_tanks_ = 1				
20 °C methylphenidate	1870	1660	7	37
20 °C ritalinic acid	–	387	1606	79
Study 2: static + biota, *N*_tanks_ = 3				
10 °C methylphenidate	187	192 ± 28	28 ± 4	122
20 °C methylphenidate	187	205 ± 19	2 ± 1	47
10 °C ritalinic acid	–	11 ± 10	202 ± 12	62
20 °C ritalinic acid	–	8 ± 2	231 ± 12	65

### Uptake in tissues of stickleback and water louse

**Fig. 2:**
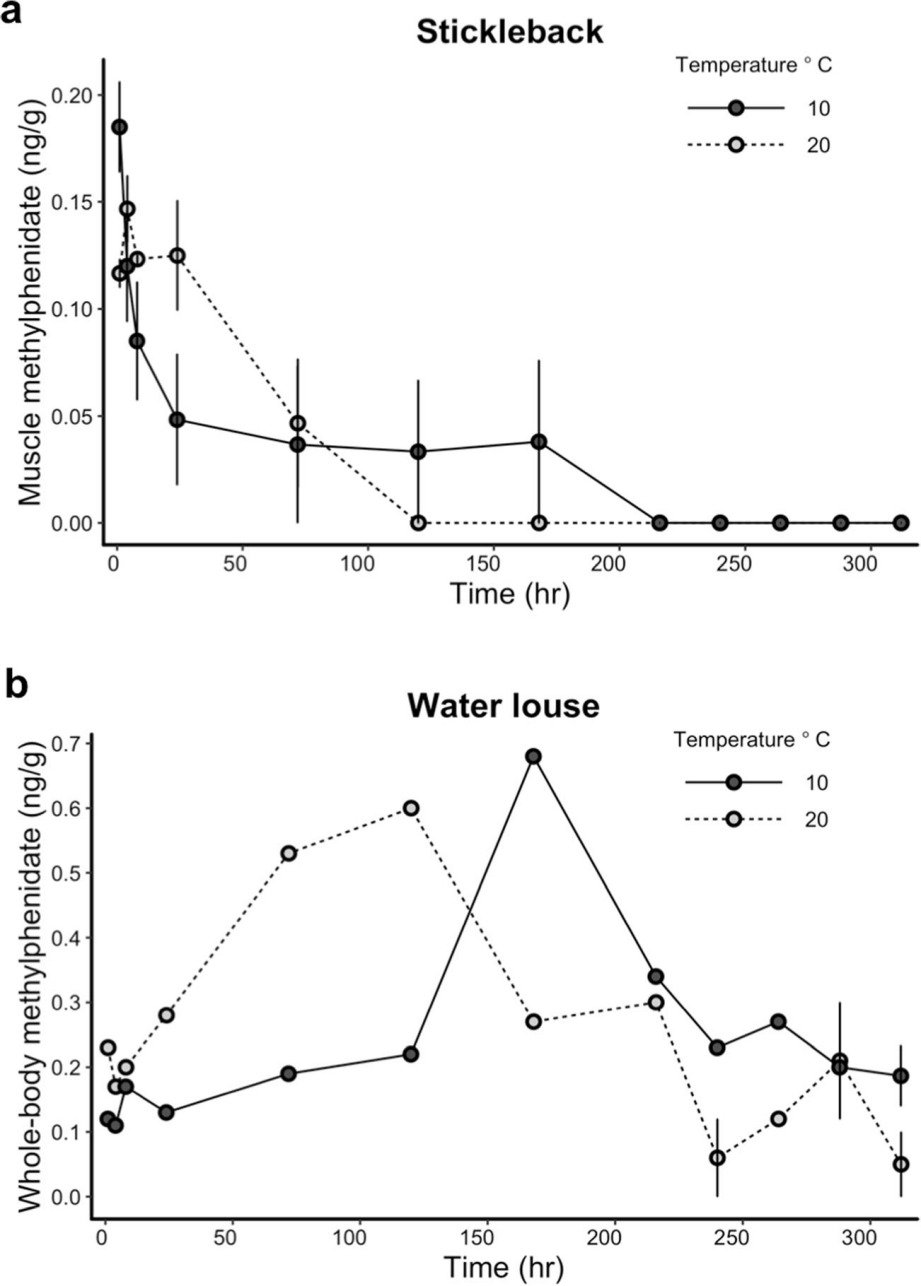
Methylphenidate concentrations in (**a**) muscle tissue from stickleback and (**b**) whole-body tissue from water louse, plotted against sampling time. Separate trend lines denote two temperature treatments. Error bars represent ± 1 standard error. Stickleback, *N*_samples_ = 3–6 per sampling time; water louse, *N*_samples_ = 1–3 per sampling time. Sample size varies due to mass of organisms collected

Stickleback and water louse took up methylphenidate in their tissues at different rates when exposed under the same experimental conditions (Fig. 2a, b). For stickleback, tissue concentrations of methylphenidate were highest within the first 4 h of exposure (Fig. 2a) and were not detectable above the LOQ on the last day of sampling. For water louse, tissue concentrations of methylphenidate peaked after 5 to 7 days (Fig. 2b), reached higher tissue concentrations than stickleback, and were still detectable at the end of the experiment (Table 2). Water louse relative tissue concentrations (ratio between tissue and water) were generally higher in the 20 °C than 10 °C treatment at the end of the experiment (Table 2, end relative tissue concentration). Neither stickleback nor water louse took up ritalinic acid above the LOQ in their tissues at any sampling point in the experiment (Table 2). See Supplementary Materials Figure S2ab for figure increased resolution of sampling across the first 24 h.

**Table 2:** Tissue concentrations (ng/g) and relative tissue concentrations (eq. 2) of methylphenidate and ritalinic acid in stickleback and water louse from study 2. Start sampling occurred 60 min after dosing. Values are shown as mean ± s.d. (range). Ranges are not given for single values. End sampling occurred on the final sampling day. *LOQ* limit of quantification, *NA* not applicable

	Start (ng/g)	Start relative tissue concentration	End (ng/g)	End relative tissue concentration
Stickleback (muscle tissue)				
10 °C methylphenidate	0.18 ± 0.05 (0.13–0.28)	1.2 ± 0.5 (0.7–2.1)	< LOQ	NA
20 °C methylphenidate	0.12 ± 0.02 (0.10–0.14)	0.5 ± 0.1 (0.4–0.7)	< LOQ	NA
10 °C ritalinic acid	< LOQ	NA	< LOQ	NA
20 °C ritalinic acid	< LOQ	NA	< LOQ	NA
Water louse (whole-body)				
10 °C methylphenidate	0.1	0.6	0.2 ± 0.1 (0.1–0.3)	7.5 ± 3.2 (4.0–10.4)
20 °C methylphenidate	0.2	1.1	0.1	50.0
10 °C ritalinic acid	< LOQ	NA	< LOQ	NA
20 °C ritalinic acid	< LOQ	NA	< LOQ	NA

## Discussion

Methylphenidate and ritalinic acid have garnered recent research attention as surface water contaminants and a potential concern for aquatic wildlife (Letzel et al. 2010; Endres et al. 2017). To support future ecotoxicology studies on these compounds, we assessed the degradation of methylphenidate and formation of ritalinic acid in the water column and tissue uptake in two aquatic organisms at two temperatures. The studies were performed well below the pKa of methylphenidate to avoid issues on ionization that has an impact on tissue uptake (Armitage et al. 2013) but also on adsorption processes (Rybacka and Andersson 2016). Methylphenidate hydrolyzed to ritalinic acid in the water column faster at 20 °C than 10 °C (37 h versus 122 h, Table 1) because higher temperatures increase the rate of the physicochemical processes leading to the transformation. Additionally, the degradation of methylphenidate to ritalinic acid was similar in exposure tanks with or without aquatic biota. The pKa of ritalinic acid is low (3.5; SciFinder) and the deprotonated form will thus dominate in our experimental setting, which typically lowers the uptake and drives the lack of uptake of this metabolite (Fu et al. 2009).

The uptake and depuration of methylphenidate in stickleback muscle tissue closely resembled that of human and rhesus macaque uptake and depuration following oral administration: a peak in plasma within 1–4 h followed by rapid depuration (Gualtieri et al. 1982; Doerge et al. 2000). Future ecotoxicological studies on methylphenidate using fish should carefully plan dosing and endpoint measurement considering compound degradation in the water column and rapid uptake in tissues. Studies to date, for example, Endres et al. (2017) and De Serrano et al. (2016), exposed fish for less than an hour and then measured physiological or behavioural endpoints. Our findings indicate that methylphenidate concentrations stay above ~ 180 ng/L (the concentration that Endres et al. (2017) found increased on zebrafish cortisol) in the water column for up to 2 days. It would be valuable to understand how methylphenidate affects fish physiology and behaviour over a longer (48 h) time-span. We assessed methylphenidate in muscle tissues, but it would also be fruitful to compare uptake across different tissue types in fish because pharmaceuticals can bioconcentrate differentially among tissues (e.g., Tanoue et al. 2015). However, in general, the low observed uptake fits well with the calculated bioconcentration factors.

In contrast, water louse did not reach peak methylphenidate concentrations until 5–7 days following the initial exposure, depending on temperature. This indicates that water louse may have different rates or mechanisms of uptake and/or different means of metabolizing or eliminating methylphenidate. In humans, methylphenidate is metabolized to ritalinic acid primarily by carboxylesterase CES1A1 (Markowitz and Patrick 2001). The role of carboxylesterases in fish and invertebrate methylphenidate metabolism (and their comparative differences) was beyond the scope of this experiment but could be a focus of future studies. Aquatic invertebrates have previously been shown to bioconcentrate certain pharmaceuticals more than vertebrates and can generally have different patterns in uptake and depuration (Meredith-Williams et al. 2012; Fong and Ford 2014; Heynen et al. 2016). For example, Lagesson et al. (2016) noted that water louse bioconcentrated higher levels of oxazepam, diphenhydramine, and hydroxyzine when compared to European perch (*Perca fluviatilis*) exposed in naturalistic pond experiment. However, these chemicals are more hydrophobic than methylphenidate which may imply more uptake via food and particulate matter. Future work should investigate any behavioural and/or physiological effects following exposure to environmentally relevant concentration of methylphenidate in aquatic invertebrates. Changes in invertebrate behaviour can have important consequences for trophic transfer of pollutants if they are more susceptible to predation (e.g., Weis et al. 2001).

In conclusion, we have provided the first analysis of the degradation and uptake of methylphenidate and ritalinic acid under controlled conditions in a geographically widespread aquatic vertebrate and invertebrate. Generally, methylphenidate appears to be of greater concern for aquatic invertebrates based on tissue uptake patterns. Concentrations of the primary metabolite, ritalinic acid, are relatively high in wastewater effluents (50–300 ng/L), but the fish and invertebrates in our study did not have detectable tissue concentrations of ritalinic acid. The above findings (e.g. half-life, tissue uptake) will be helpful for designing future ecotoxicological studies investigating the biological impacts (e.g. behaviour, physiology) of methylphenidate and ritalinic acid in aquatic systems.

## Electronic supplementary material

ESM 1 (DOCX 5534 kb)
