# Supplementary material for: Stability and uptake of methylphenidate and ritalinic acid in nine-spine stickleback (Pungitius pungitius) and water louse (Asellus aquaticus)
Source: Environ Sci Pollut Res Int. 2019 Feb 25;26(9):9371–8. doi: 10.1007/s11356-019-04557-9 (PMC6469618; doi:10.1007/s11356-019-04557-9)
Supplement: Supplementary file 1 — (DOCX 5534 kb) [file 11356_2019_4557_MOESM1_ESM.docx]

**Stability and bioconcentration of methylphenidate and ritalinic acid in nine-spine stickleback (*Pungitius pungitius*) and water louse (*Asellus aquaticus*)**

**Erin S. McCallum, Richard H. Lindberg, Patrik L. Andersson, Tomas Brodin**

**Supplementary Methods and Results**

*QA/QC methods*

Extraction efficiency of the analytes (methylphenidate and ritalinic acid) from non-exposed stickleback and water louse samples were made by pre-extraction fortification (5 ng) of the target compounds to each sample matrix (n=5, average weight 0.08 g). The internal standard (IS) methylphenidate-D9 (5 ng) was added to the sample extracts before instrumental analysis to allow the determination of analyte/IS area ratio. Analyte/IS area ratios from these samples were compared to the equivalent in matrix-matched standards (n=3), post-extraction fortified (5 ng of analytes and IS), in order to calculate recoveries without the influence of potential matrix ion signal suppression. Ion suppression was determined by the comparison of the analyte and IS peak areas in matrix-matched standards to methanol-based standards.

*QA/QC results*

For the stickleback samples, the recoveries of methylphenidate and ritalinic acid were 100 ± 6% and 63 ± 4%, respectively. Similar recoveries, 102 ± 4% and 75 ± 6%, were obtained for the two analytes in the water louse samples. Both of the analytes and the IS were influenced by matrix ion signal suppression and were as follows in the stickleback and water louse sample extracts (average): ritalinic acid, 62% and 48%; methylphenidate, 39% and 33%; and methylphenidate-D9, 51% and 42%.

**Supplementary Figures**


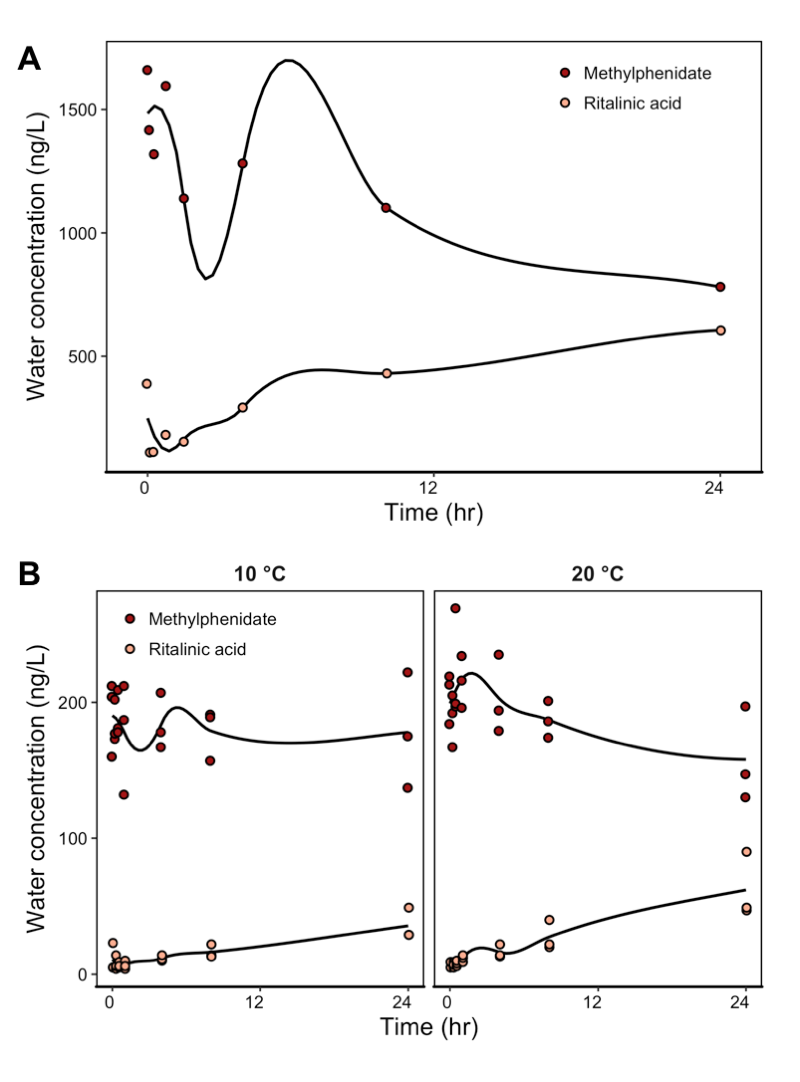


**Figure S1:** Truncated data from Figure 1 to better show time course of the first 24 hours of sampling. Concentrations of methylphenidate and ritalinic acid in exposure aquaria under **(A)** static conditions with no biota (N*_tanks_* = 1), and **(B)** static conditions with biota, facetted by temperature treatment (N*_tanks_* = 3). Each point represents one sample. Solid lines represent the mean fit by a loess curve.


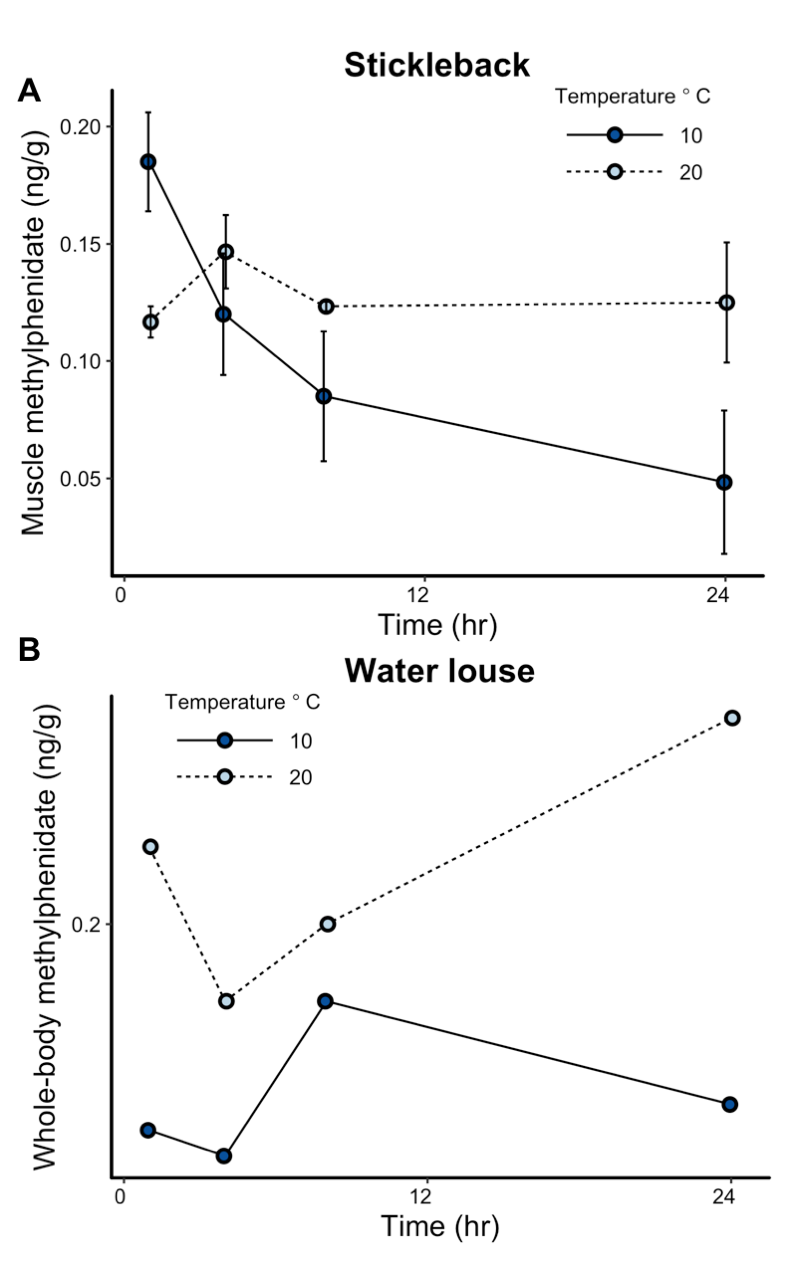


**Figure S2**: Truncated data from Figure 2 to better show time course of the first 24 hours of sampling. Methylphenidate concentrations in **(A)** muscle tissue from stickleback, and **(B)** whole-body tissue from water louse, plotted again sampling time. Separate trend lines denote two temperature treatments. Error bars represent ± 1 standard error. Stickleback, N_samples_ = 3 - 6 per sampling time, water louse, N_samples_ = 1-3 per sampling time, sample size due to mass of organisms collected.
